# Cooperative spatial modelling of hospital compliance with minimum caseload requirements

**DOI:** 10.1186/s12942-025-00442-6

**Published:** 2026-01-27

**Authors:** Limei Ji, Max Geraedts, Werner de Cruppé

**Affiliations:** Institute for Health Services Research and Clinical Epidemiology, Philipps- Universität Marburg, Karl-von-Frisch-Strasse 4, Marburg, 35043 Germany

**Keywords:** Regional cooperation, Minimum caseload requirements, German hospital quality reports, Decision space, Exceptional permission, Regionalised health services policy, Spatial modelling

## Abstract

**Background:**

Minimum caseload requirements (MCRs) ensure medical treatment quality but may negatively affect spatial accessibility to health care. Previous studies optimised the caseload distribution via spatial models, with a focus on balancing spatial concentration and accessibility with centralised case redistribution models. This study seeks to capture hospitals as active participants in MCR policy decisions by incorporating their intentions and motivations regarding MCRs within their spatial context, considering current caseloads and neighbouring hospital distances.

**Methods:**

The study modelled four MCR procedures separately in an individual model in accordance with the German policy context: complex oesophageal interventions, complex pancreatic interventions, stem cell transplantation, and total knee replacement. The spatial model for Germany involved three steps: (1) delimiting cooperating hospitals, (2) iterative grouping, and (3) categorising hospital groups.

**Results:**

The grouping process described above resulted in 55 (oesophagus), 126 (pancreas), 39 (stem cell), and 672 (knee) groups across Germany. A total of 50.9%, 49.2%, 51.3%, and 81.5% respectively of these groups contained only one hospital (no cooperation needed). 7, 28, 2, and 22 groups require joint MCR compliance, whereas 19, 21, 8, and 8 hospitals are recommended special permission with a reduced caseload threshold to ensure spatial accessibility for certain regions. The results inform regional policy makers based on the hospital decision space.

**Conclusions:**

This study models potential hospital cooperation based on proximity and caseload, introducing joint MCR compliance. The modelling process supports the formation, categorisation, and analysis of hospital groups, with parameter thresholds enabling flexible policy testing. This approach considers hospitals’ intentions and motivations regarding MCRs, and reserves their decision space. This spatial model provides a theoretical basis for granting exceptional MCR permissions to improve spatial accessibility.

**Supplementary Information:**

The online version contains supplementary material available at 10.1186/s12942-025-00442-6.

## Background

The quality of medical treatment is a key focus in health services research. Minimum caseload requirement (MCR) is one of the related policy concepts, which is based on evidence from studies and systematic reviews demonstrating a volume‒outcome relationship: hospitals treating a higher number of cases per year achieve better treatment quality, characterized by lower complication rates and reduced mortality across a wide range of medical procedures [1–6]. MCR policies have already been implemented in several countries [7–11]. MCR requires hospitals to perform annually a minimum number of specific procedures that have demonstrated volume‒outcome‒relationships. The implementation of MCRs aims to concentrate cases regionally in fewer hospitals with a higher number of cases, which meet the required caseload thresholds and are therefore expected to provide higher treatment quality. However, limiting treatment provision to a smaller number of hospitals may worsen access to health care services [12–15]. 

Several studies have analysed the geographical accessibility of health care facilities, employing spatial models to simulate and optimise caseload distributions by incorporating factors such as travel time and accessibility, geographical coverage of hospitals, hospital caseload and capacity, patient choices, and related uncertainties [16–19]. Various policy objectives are addressed, including regional hospital capacity planning [20, 21], ensuring accessibility [22, 23], equity [24], and the MCR [25–31].

MCR policy-related models simulate case relocation dynamics from noncompliant hospitals to those which meet MCR requirements. In this study, we refer to these as ‘case relocation models’. These models include the initial distribution of case numbers, patients’ hospital selection (patient redistribution logic, considering factors such as travel time), and the service closure logic of hospitals [25–31]. Iterative modelling design is commonly used for simulating these dynamics. Changes in accessibility after ‘service closure’ are assessed via statistics of linear distance [32], average driving time [26, 28, 29, 31, 33–36], isochrone maps [27], or the identification of hot and cold spots of accessibility indices [37]. In the case relocation models, patients select hospitals based on the principle of nearest distance or shortest driving time, while hospitals accept patients passively. To prevent extreme modelling outcomes (e.g., extensive areas without MCR services or excessively high caseloads concentrated in certain hospitals), additional constraints are introduced as model parameters to evaluate potential policy adaptations. For example, the study by Mennicken et al. [25] and the study by Vogel et al. [30] simulate their scenarios of MCR implementation using a service closure logic, which is influenced not only by a fixed minimum caseload threshold but also by additional factors, such as regional travel time thresholds or hospital capacity thresholds. These differentiated MCR variants aim to address regional disparities in health care accessibility.

All the above-mentioned case relocation modelling and optimisation settings operate under a unified set of case redistribution rules, which are interactive but centrally governed, and reflecting a centralised case redistribution model with a single decision-maker structure. These approaches align with the scenarios where health care policy is designed and operated by a ‘national’ decision-maker. Even in models that incorporate additional factors – such as travel time thresholds and hospital capacity constraints – these thresholds are typically regulated at a unified national level in practice. In contrast, the World Health Organisation (WHO) advocated a multilevel approach to health care system planning, distinguishing between strategic planning and operational planning. It emphasises the importance of involving health care facilities in formal organisational activities, ensuring facilities’ decision space to operate within the planning process [38]. From the theoretical aspect, redistribution models typically assume that hospitals accept patients passively according to the regulations – whether based on minimum caseload threshold, maximum travel time limits, or capacity constraints – and do not participate in MCR policy decisions. To account for the expertise of procedure-delivering specialists and to incorporate the ‘intentions’ and ‘motivations’ of hospitals, this study goes beyond the deterministic modelling of case redistribution and considers hospitals as active participants in planning and market dynamics. Hospital compliance or withdrawal from MCR can be represented through their interrelationship with neighbouring hospitals. In commonly used relocation models, these interrelationships are defined mainly by competitive case redistribution. However, they may also involve hierarchical structures, dependency relations, or cooperative networks [39–41].

In this study, we construct a key metric—the total caseload of proximate hospitals (hereafter referred to as regional hospital groups)—and compare it against the MCR thresholds. By analysing both the group-level total caseload relative to MCR thresholds and the internal distribution of caseloads within each group, we assess the feasibility and challenges hospitals may face when meeting MCR thresholds individually, including scenarios where cases may be shared or transferred among neighbouring hospitals.

In addition, this modelling framework enables the examination of an additional adaptation of MCR—the possibility of joint compliance with MCR thresholds. In this context, the total caseload within groups also captures cooperative spatial interrelationships between hospitals and reflects the potential for regional hospital cooperation. Unlike classical case redistribution models, in which hospitals’ intentions and motivations are explicitly encoded into model dynamics, our study treats their intentions and motivations analytically.

## Methods

In contrast to commonly used redistribution models of MCR that treat hospitals individually, the current spatial model introduces a grouping step and demonstrates its application using hospital distribution data from Germany. This step identifies cooperating hospital groups, and allocates decision space, defined here as the agency of hospitals to evaluate trade-offs and actively decide on compliance with or withdrawal from MCR through internal cooperative arrangements. By considering the total group caseloads and its internal distribution, the model assesses potential intentions, and facilitates co-decisions or joint compliance with MCRs, meanwhile providing recommendations for MCR-related cooperation. In this way, the concept of joint compliance is introduced as a new option. The approach is outlined in the following steps.

(1) Delimitation of cooperating hospitals: The spatial modelling establishes a baseline exclusion threshold for hospitals with historically very low caseloads (occasional surgery) or caseloads significantly below MCR compliance. Hospitals above this threshold are referred to as cooperating hospitals, which can include both MCR-compliant and noncompliant hospitals. Within our model, these hospitals participate in a cooperative case distribution process and are represented as action agents with their decision space.

(2) Iterative grouping process: During the modelling process, these cooperating hospitals are no longer excluded from the process, regardless of their individual compliance with MCRs. Instead, hospitals are now grouped based on their distance matrix, which consists of two types of distances: straight-line distance and driving time, and the total caseload of each group. The focus then shifts to evaluating the grouping results and the decision space available for MCR cooperation.

(3) Categorisation of hospital groups: The grouping results are analysed in two dimensions: the grouping distance and the group caseload. These two dimensions influence the decision space and the subsequent recommendations.

### Data sources and MCR-related key metrics

MCR caseloads were extracted from German Hospital Quality Reports (GHQRs) 2016–2021 [42], which have been validated and longitudinally linked based on prior research by Ji et al. [43, 44]. The 6-year caseloads are weighted and aggregated to estimate an expected caseload for one year (see Table 1 for the weighting formula). The weighting follows the principle that the latest three years contribute more to the expectation than the earlier three years. The hospital addresses provided in the GHQR are converted into geocodes via the Google API geocoding function [45]. The distances between hospitals were measured in two ways: straight-line distances were calculated using GeoPy [46], and driving times were estimated using OpenRouteService [47]. The iterative modelling was conducted in Python 3.10. The zoning and maps were calculated and visualised with ArcGIS Pro 2024 [48].

In 2004 and 2006, six MCR interventions were introduced in Germany, including complex oesophageal and pancreatic interventions, stem cell transplantation, total knee replacement, liver transplantation, and kidney transplantation. The MCR for liver and kidney transplantation are excluded from this study because they are highly concentrated, involving approximately 20 and 40 hospitals nationwide, respectively, which are constantly compliant with the MCR [7]. Four MCR procedures were modelled separately in an individual model: complex oesophageal interventions (since 2021, 26 cases per year, previously 10 cases per year), complex pancreatic interventions (since 2022, 20 cases per year, previously 10 cases per year), stem cell transplantation (since 2023, 40 cases per year, previously 25 cases per year), and total knee replacement (since 2006, 50 cases per year) [49]. Since the aim of this study is to observe and evaluate the future- and practice-oriented implications of the MCR policy, including potential changes resulting from the updated thresholds, we used the currently valid (new) thresholds, even though the caseload data predate the implementation of these new thresholds.

**Table 1:** Iterative grouping process: model parameters

Model parameter		Oesophagus	Pancreas	Stem cells	Knee
Hospital expected case number		(caseload 2016 + caseload 2017 + caseload 2018 + caseload 2019 × 2 + caseload 2020 × 2 + caseload 2021 × 2)/9			
MCR-threshold		26	20	40	50
1/2 MCR-threshold		13	10	20	25
For round 0		active (searching) hospitals: caseload < 1/2 MCR-threshold (noncooperating hospitals), passive (being searched) hospitals: caseload ≥ 1/2 MCR-threshold (cooperating hospitals)			
For round 1		active (searching) hospitals and passive (being searched) hospitals: caseload ≥ 1/2 MCR-threshold (cooperating hospitals)			
From round 2 onwards		active (searching) hospitals: caseload < MCR-threshold (cooperating hospitals), passive (being searched) hospitals: caseload ≥ MCR-threshold (cooperating hospitals)			
Searching distance in round 1: max driving duration in minutes/max straight-line distance in km		20 min/10 km	20 min/10 km	30 min/20 km	10 min/0 km
Searching distance’s extending step: max driving duration in minutes/max straight-line distance in km		5 min/5 km	5 min/5 km	5 min/5 km	5 min/5 km
Area percentage by accessibility	20 min	14.2%	33.6%	10.7%	68.9%
	40 min	63.6%	87.3%	55.5%	98.8%
	60 min	93.3%	98.8%	89.0%	99.8%

### Delimitation of cooperating hospitals

**Fig. 1:**
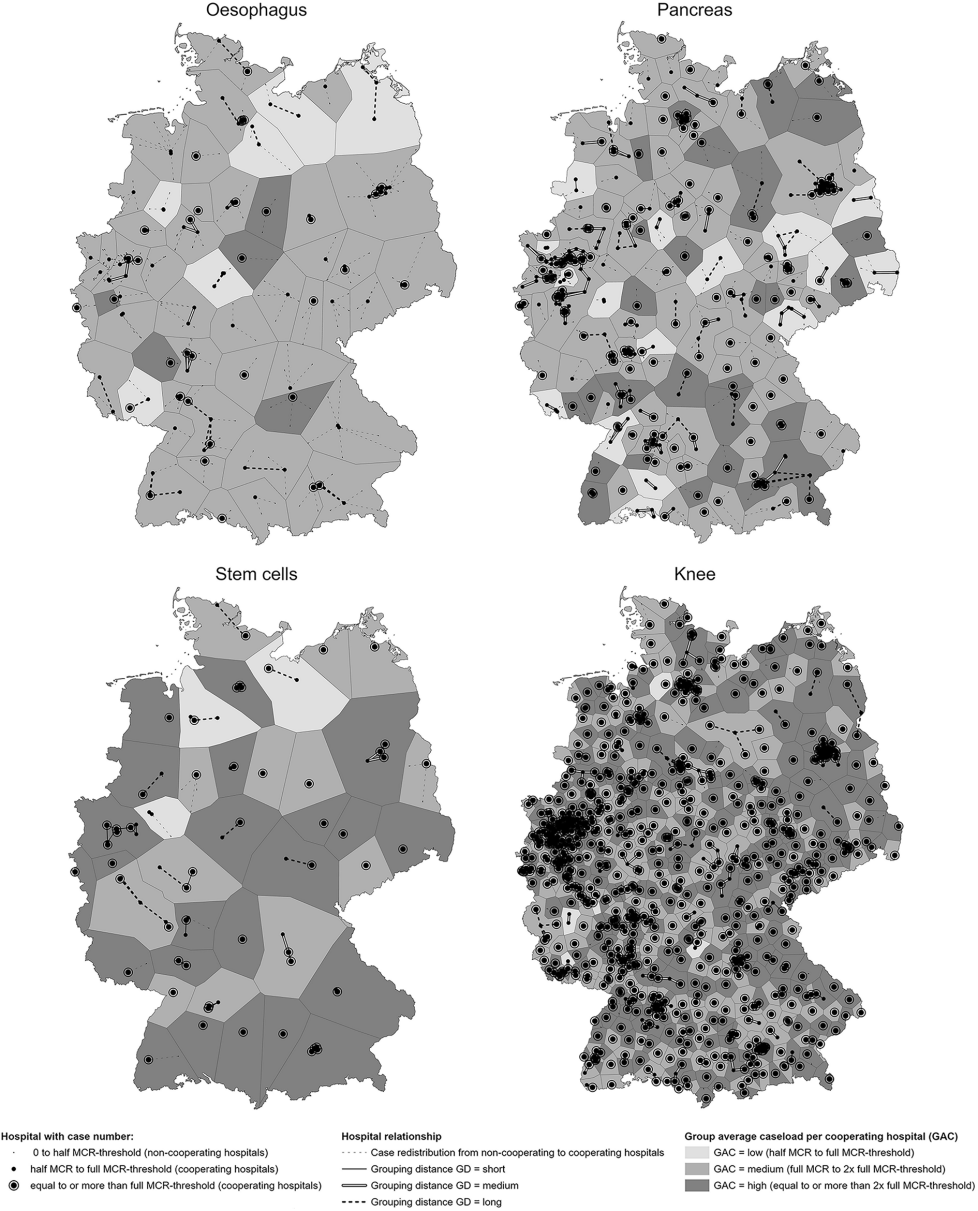
Hospital grouping regions illustrated by aggregated hospital case numbers (2016–2021) and their spatial interrelationships. Aggregated **hospital case number** 2016–2021 = (caseload 2016 + caseload 2017 + caseload 2018 + caseload 2019 × 2 + caseload 2020 × 2 + caseload 2021 × 2)/9. **The MCR thresholds were as follows**: oesophagus, 26 cases/year; pancreas, 20 cases/year; stem cells, 40 cases/year; and knee, 50 cases/year. **Critical proximity boundary near and far** in this study: oesophagus: 20 min/10 km and 35 min/25 km, pancreas: 20 min/10 km and 35 min/25 km, stem cells: 30 min/20 km and 40 min/30 km, knee: 20 min/10 km and 30 min/20 km. The German administrative base map was obtained from GADM (Version 2.5, July 2015) [50]

**Fig. 2:**
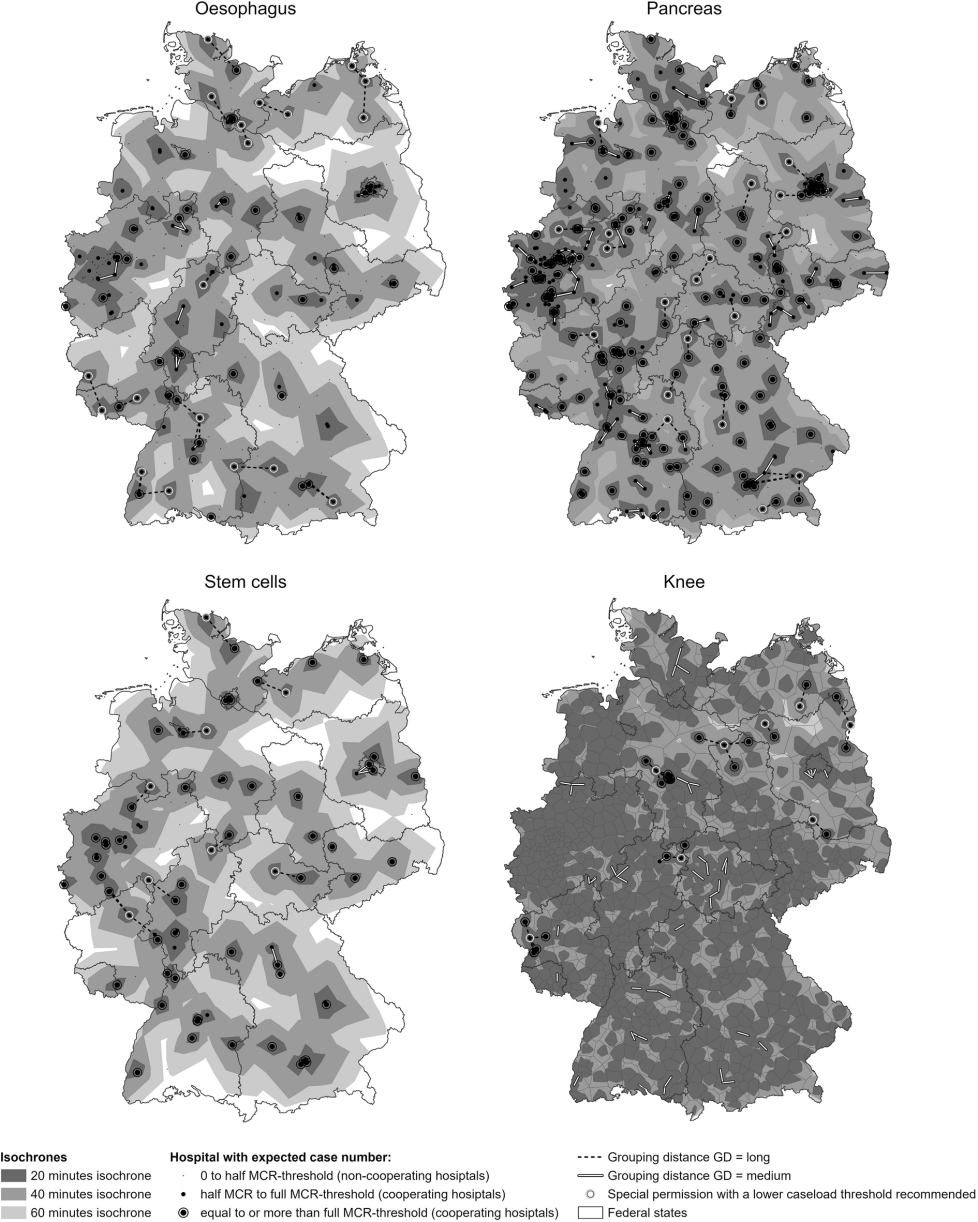
20-, 40- and 60-minute isochrones for cooperating hospitals performing oesophagus, pancreas, stem cells and knee procedures. Owing to the high density of hospitals for knee procedures, only those with long grouping distances are shown for clarity. Isochrones are calculated via OpenRouteService’s isochrone function, with hospitals set as destinations and a smoothing factor of 100. The German administrative base map was obtained from GADM (Version 2.5, July 2015) [50]

Hospitals with a caseload ≥ 1/2 of the MCR threshold are defined as our research objects (the baseline inclusion threshold) and are referred to as **cooperating hospitals** (see ‘half MCR to full MCR threshold’ and ‘equal to or more than full MCR threshold’ in Figs. 1 and 2). These hospitals have already achieved a certain level of experience with the relevant MCR procedures, as indicated by their caseloads, and will not be excluded from modelling. Hospitals with caseloads below the 1/2 MCR threshold are defined as **noncooperating hospital**s (see ‘0 to half MCR threshold’ in Figs. 1 and 2). The caseloads of noncooperating hospitals are relocated to cooperating hospitals based on the nearest straight-line distance (see ‘round 0’ in the following section, ‘Iterative grouping process’). The accessibility of the cooperating hospitals is illustrated on the maps, with their reachable areas within 20, 40, and 60 min indicated (see Fig. 2). Within 60 min, 93.3% (oesophagus), 98.8% (pancreas), 89.0% (stem cells), and 99.8% (knee) of the country is covered by cooperating hospitals (see Table 1).

### Iterative grouping process

**Fig. 3:**
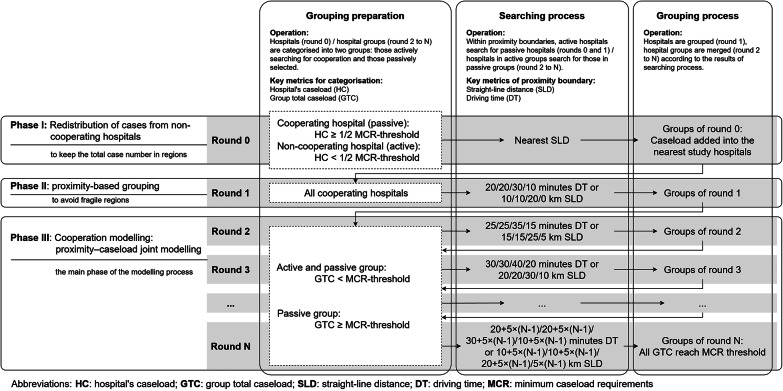
Iterative calculation process: Grouping hospitals to reach the MCR threshold jointly

The iterative grouping process is a modelling approach that is structured in successive rounds. Hospitals are represented by their geographic coordinates and treated as points on the map, with attributes of MCR caseload, straight-line distance, and driving time to other hospitals. During the modelling process, each hospital (point) searches for partners, if necessary, that is, when its caseload does not meet the MCR threshold. The search process unfolds in rounds, with the search radius expanding in each subsequent round. If a hospital successfully finds partner hospitals, they form a group. If the joint MCR caseload of the group still falls short of the MCR-threshold, the hospitals continue searching for additional partners. Once the MCR-threshold is met, the group terminates its search. The grouping process consists of three main phases: Phase I — redistribution of cases from non-cooperating hospitals; Phase II — proximity-based grouping; and Phase III — proximity–caseload joint modelling (as shown in Fig. 3).

In **Phase I** (**round 0)**, the expected cases from noncooperating hospitals are redistributed to the nearest cooperating hospitals based on straight-line distance (see ‘round 0’ in Fig. 3). This reflects the baseline inclusion process, which involves determining which hospitals participate in the modelling. This is the only step in this study involving case redistribution. From round 1 onwards, caseloads serve as a characteristic of the hospital and hospital group in the modelling. The redistribution in round 0 supports maintaining the overall caseload across the country.

In **Phase II** (**round 1)**, cooperating hospitals were grouped on the basis of proximity alone, disregarding caseloads, to avoid excessive spatial fragmentation in the grouping results when hospitals are very close to each other. Proximity is defined as the distance between cooperating hospitals below a specified **proximity boundary**. If hospitals are grouped, this distance is referred to as the **grouping distance (GD)**. Two metrics are used to define the grouping distance and proximity boundary: straight-line distance (in kilometres) and driving time (in minutes). Hospitals are grouped if either the straight-line distance or the driving time falls within the proximity boundary. The straight-line distance represents general proximity, accounting for various transportation modes, such as walking, cycling, bus, car, or helicopter ambulances, whereas the driving time is specific to more common transportation modes, such as car or ambulance transport.

For round 1, the proximity boundaries are set as follows: 20 min or 10 km for the oesophagus and pancreas procedures, 30 min or 20 km for the stem cell procedures, and 10 min or 0 km for the knee, where the 0 km threshold indicates that straight-line distance is exceptionally not considered in this round (Table 1). The proximity boundary value pairs are selected based on the linear correlation between the driving time and straight-line distance within the current regional distribution of hospitals. (See Figure S1 in the supplementary materials). The variation in boundary definitions in round 1 across four MCR procedures arises from differences in the basic spatial density of hospitals providing these MCR procedures.

In **Phase III** (from **round 2** to the final **round N)**, the iterative grouping of hospitals constitutes the main phase of the modelling process. Before each round, the **group total caseload (GTC)** was calculated as an indicator of whether the group jointly meets the MCR threshold (see Fig. 3). Groups with a GTC below the MCR threshold are referred to as **active groups**. Hospitals in these active groups must continue searching for additional grouping partners and can also be selected as partners either by other active groups or passive groups. The **passive groups** meet the MCR threshold jointly, including single hospitals that comply individually.

In the iterative grouping process from round 2 to round N, hospitals in active groups search for partners from both active and passive groups within the proximity boundary. If successful, the active and selected hospitals, along with their respective groups, are merged into a new group. The corresponding straight-line distance, driving time, and round number are the three key metrics used to describe the **grouping distance (GD)**. The proximity boundaries expand incrementally in each round by 5 km for straight-line distance and 5 min in driving time, based on the initial proximity boundary set in round 1 (see Fig. 3; Table 1). This stepwise extension allows the selection of multiple hospitals in a single round. The selection of the initial search range and the extension steps align with the observed inter-hospital distances for the four MCR procedures, as illustrated in Figure S1 in the supplementary materials. These parameters were calibrated to balance the grouping process, ensuring that each step typically aggregated a small number of hospitals – neither too many at once nor none for extended sequences of steps. The extension steps correspond to decreasing degrees of priority or feasibility for grouping.

If all groups’ GTCs meet the MCR threshold, the regional hospital grouping process is completed. The resulting groups represent the smallest regional units capable of jointly reaching the MCR threshold through cooperation, implying that at least the last hospital in each group must cooperate with others to achieve the MCR threshold. The grouping results indicate the necessity of hospital cooperation for joint MCR compliance. In section ‘Categorisation of hospital groups’, the feasibility of the grouping results will be analysed.

To enhance the visibility of the modelling results, zoning was constructed via the Thiessen polygon process in ArcGIS Pro 2024 [48] to present the hospital groups (see the zoning in Fig. 1). This zoning estimates the geographical coverage of health service provision by hospitals geometrically, without taking into account factors such as population distribution or traffic conditions. As MCRs explicitly refer to elective interventions associated with a limited proportion of emergencies, the modelling process does not use emergencies as a modelling parameter.

### Categorisation of hospital groups

The feasibility of cooperation among hospitals within a hospital group is determined by two dimensions: group caseload richness and distance friction. Both dimensions together define the feasibility of the decision space for each hospital or cooperation region. A two-dimensional categorisation is illustrated in a coordinate grid-box system. The final decision on hospital grouping is made within this decision space by hospitals, incorporating each hospital’s preferences based on individual factors, such as development strategies, the interplay of difficulty (or, conversely, feasibility), and individual hospital preferences.

**Fig. 4:**
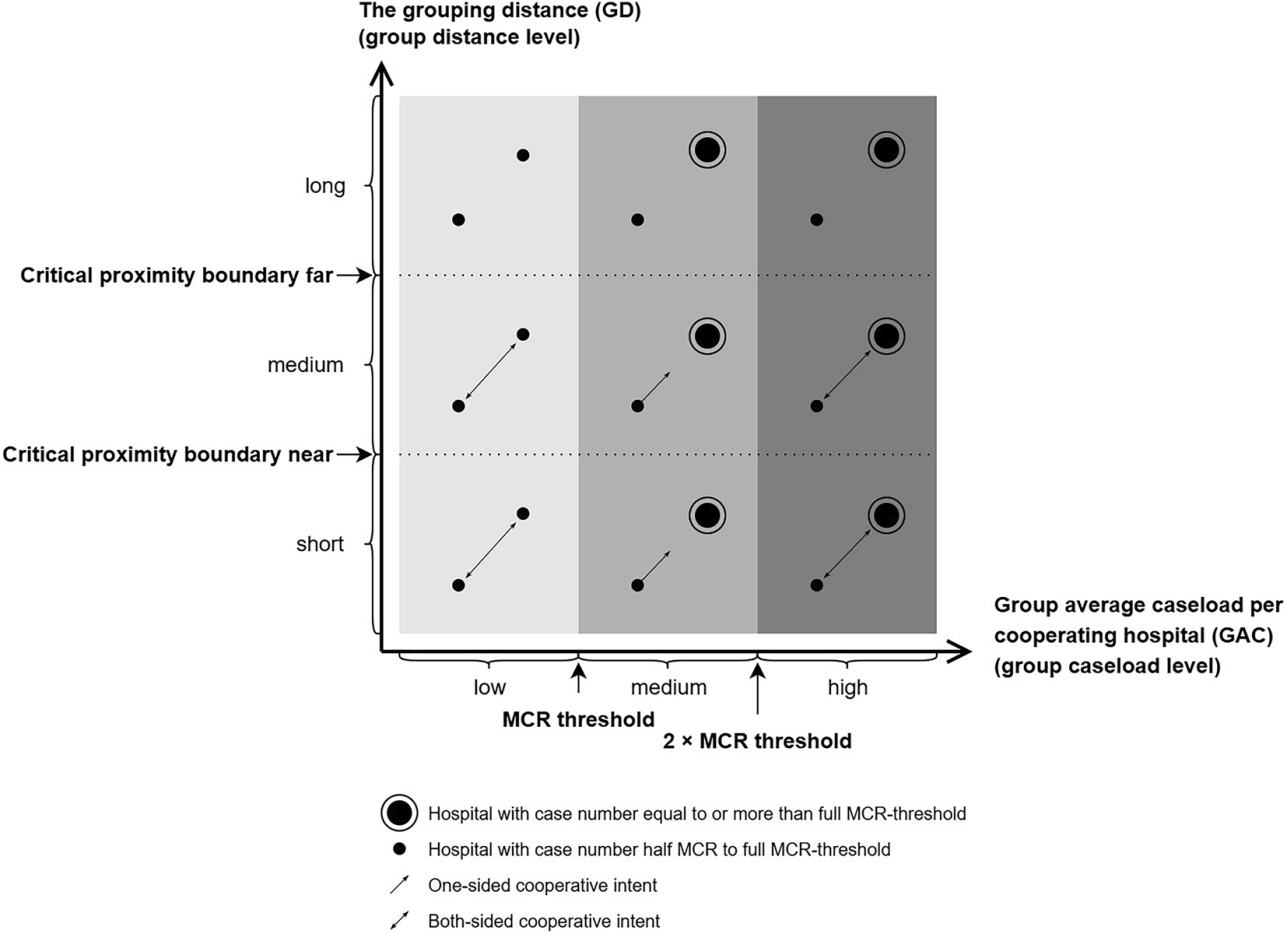
Hospital group characteristics, intention and motivation analysis

Hospital groups are categorised via a 9-box grid, with the **group caseload level** on the horizontal axis and the **group distance level** between hospitals on the vertical axis (see Fig. 4). The group caseload level, represented by the **group average caseload per cooperating hospital in the group (GAC)**, reflects the case density relative to the existing number of cooperating hospitals per group, which implies the average ease of a group meeting the MCR. The GAC is divided into three levels: (1) low: GAC below the MCR threshold, indicating that the caseload is just sufficient for MCR compliance; (2) medium: GAC between the MCR threshold and twice the MCR threshold; and (3) high: GAC exceeding twice the MCR threshold, representing more than a sufficient caseload for MCR compliance (see Fig. 4; Table 2 for the respective GAC thresholds). The group distance level, represented by the **grouping distance** (GD), reflects the distance friction of caseload sharing. The distance level is also divided into three categories, short, medium, and long, based on two self-defined **critical proximity boundaries: near and far** (see Fig. 4; Table 2 for the respective critical proximity boundaries). The ‘critical proximity boundary: near’ defines the feasible distance for ‘patient relocation’ as feasible or not (feasible if GD < ‘critical proximity boundary near’; not feasible if GD ≥ ‘critical proximity boundary near’). The ‘critical proximity boundary: far’ defines the feasible distance for ‘hospital cooperation’ (feasible if GD < ‘critical proximity boundary far’; not feasible if GD ≥ ‘critical proximity boundary far’). The critical proximity boundaries in this model can be adjusted according to policy needs.

**Table 2:** Thresholds and critical values for 9-grid box analysis

	Oesophagus	Pancreas	Stem cells	Knee
MCR threshold	26	20	40	50
Twice the MCR threshold	52	40	80	100
Critical proximity boundary near	up to round 1 (< 20 min/10 km)	up to round 1 (< 20 min/10 km)	up to round 1 (< 30 min/20 km)	up to round 3 (< 20 min/10 km)
Critical proximity boundary far	from round 5 (≥ 35 min/25 km)	from round 5 (≥ 35 min/25 km)	from round 4 (≥ 40 min/30 km)	from round 6 (≥ 30 min/20 km)

**Table 3:** Hospital group characteristics analysis and recommendations for action

Category		Recommendation for action, considering area-comprehensive service provision and sharing overload
Grouping distance (GD)* = long		
The long GD hinders the relocation of cases and the hospital cooperation		To ensure comprehensive service provision, it is necessary to retain even noncompliant hospitals in the area. Special permission for **individual** low-caseload hospitals is recommended to ensure the provision of area-comprehensive services
Grouping distance (GD)* = medium		
With the medium GD, the relocation of patients is expected to be challenging, but hospital cooperation and sharing of experience are possible		To facilitate the patients with area-comprehensive service provision, noncompliant hospitals can be retained and cooperate with grouping hospitals for experience sharing. Special permission for group **joint** compliance is recommended
Grouping distance (GD)* = short		
Due to the short GD, both patient relocation and hospital cooperation are expected to be straightforward		The noncompliant hospitals will either withdraw services or share the overload of high-caseload hospitals under group joint compliance. For sharing the overload, special permission for group **joint** compliance is recommended. Otherwise, no additional policy changes are needed

*GD: grouping distance. long: GD ≥ critical proximity boundary far; medium: critical proximity boundary near ≤ GD < critical proximity boundary far; short: GD < critical proximity boundary near

The categorisation based on group caseload levels and distance levels reflects varying feasibility of sharing cases and achieving the MCR threshold, either individually or jointly (see Fig. 4; Table 3). Hospital groups with long GDs exhibit high distance friction, making case sharing (patient relocation) and experience sharing (hospital cooperation) difficult. To ensure the spatial accessibility of service provisions under long GD, it may be advisable to grant special performance permissions to individual hospitals (without requiring cooperation with other hospitals) for MCR procedures with a lower caseload threshold. This recommendation for special permission is comparable with the official MCR’s exceptional permissions granted to ensure the spatial accessibility of service provision. A grouping distance of medium GD indicates that hospital cooperation is straightforward, but patient relocation is challenging. In these cases, joint compliance with MCRs is recommended to ensure the spatial accessibility of service provisions. This joint compliance is driven primarily by noncompliant hospitals, as well as compliant hospitals, which may seek support from other hospitals to share the overload (see arrow patterns in Fig. 4). When hospital groups have grouping distances below the ‘critical proximity boundary near’, both case sharing and patient relocation are considered feasible. Based on their needs, noncompliant hospitals may either share overload cases with compliant hospitals or collaborate with other noncompliant hospitals to achieve joint MCR compliance. If neither approach is feasible, these hospitals are required to withdraw services under the current MCR regulations. Figure 4; Tables 2 and 3 support the interpretation of Figs. 1 and 2.

## Results

The iterative grouping process continued up to rounds 10, 9, 8, and 10 for the oesophagus, pancreas, stem cells and knee MCR procedures, respectively. This corresponded to the largest grouping distances of 65 min/55 km, 60 min/50 km, 65 min/55 km, and 55 min/45 km until the group total caseload (GTC) for all groups met the MCR threshold (see Table 4). The cooperation modelling results yielded a total of 55, 126, 39, and 672 hospital cooperation groups, respectively. All groups met the MCR thresholds through their group total caseloads (GTCs), indicating joint compliance, although not necessarily their group average caseloads (GACs), which reflect hospital individual compliance within their group (see Table 4). A total of 50.9%, 49.2%, 51.3% and 81.5% of these groups consisted of only one cooperating hospital. These groups cover 34.6%, 35.0%, 39.2% and 71.0% of the country, respectively (see Fig. 2; Table 4). The frequency distribution of hospital groups across the 9 grid box categories are listed in Table 4.

Under the parameters set in this study, the number of hospitals (with long GD) recommended for special permission with a reduced caseload threshold to ensure the spatial accessibility of service provision are as follows: 19 for the oesophagus, 21 for the pancreas, 8 for stem cells, and 8 for knee procedures (see Fig. 2; Table 4). The total numbers of groups with at least one medium GD, indicating a need for joint MCR compliance, are 7 (12.7%), 28 (22.2%), 2 (5.1%), and 22 (3.3%), respectively. Additionally, the groups with potential overload (characterised by high GAC) and the need for sharing cases are depicted in Fig. 1.

**Table 4:** Statistics of Cooperation groups and regions: area, group case number, number of cooperating hospitals, and accessibility

		Oesophagus	Pancreas	Stem cells	Knee
The last round number		Round 10 (60–65 min/ 50–55 km)	Round 9 (55–60 min/ 45–50 km)	Round 8 (60–65 min/ 50–55 km)	Round 10 (50–55 min/ 40–45 km)
Number of groups		55	126	39	672
Group total caseload (GTC)	Min	27.89	20.00	47.22	50.00
	Median	51.44	42.11	192.00	124.39
	Avg.	81.20	96.33	207.79	208.82
	Max	371.00	1413.67	829.78	3582.00
Group average caseload per cooperating hospital (GAC)	Min	13.85	13.41	23.61	30.56
	Median	37.10	27.50	89.67	110.61
	Avg.	42.91	33.35	112.97	145.10
	Max	236.11	104.22	272.78	1190.56
Number of groups with grouping distance (GD) categorised as	Only near	9 (16.4%)	26 (20.6%)	9 (23.1%)	95 (14.1%)
	Only medium	2 (3.6%)	9 (7.1%)	0 (0.0%)	16 (2.4%)
	Only far	7 (12.7%)	5 (4.0%)	4 (10.3%)	5 (0.7%)
	A mixture of near and medium	4 (7.3%)	11 (8.7%)	2 (5.1%)	5 (0.7%)
	A mixture of near and far	4 (7.3%)	5 (4.0%)	4 (10.3%)	2 (0.3%)
	A mixture of near, medium and far	1 (1.8%)	5 (4.0%)	0 (0.0%)	0 (0.0%)
	A mixture of medium and far	0 (0.0%)	3 (2.4%)	0 (0.0%)	1 (0.1%)
Percentage of groups with only one cooperating hospital*		28 (50.9%)	62 (49.2%)	20 (51.3%)	548 (81.5%)
Percentage of area in regions with only one cooperating hospital*		34.6%	35.0%	39.2%	71.0%
Number of hospitals recommended for special permission with a reduced caseload threshold to ensure area-comprehensive service provision		19	21	8	8

*A cooperating hospital refers to a hospital with case number more than half MCR

The final hospital groups and their corresponding regions are presented in Fig. 1, while the accessibility of the cooperating hospitals is displayed in Fig. 2. The grouping distances for ‘medium GD’ and ‘long GD’, represented by thick solid white lines and thick dashed lines, are consistent in both Figs. 3 and 4. The thin solid lines for short GDs are often less visible due to their limited length. Figure 2 shows the boundaries of the federal states to provide additional context for the interpretation of the modelling results. The relevant statistics are provided in Table 4. Approximately half of the groups for oesophagus, pancreas, and stem cells, as well as 81.5% of the groups for knee interventions, have only one cooperating hospital within the region. These hospitals cover their respective areas with cases compliant to MCR, and therefore do not require cooperation or face competition from other hospitals in the same region. Such single-hospital groups account for 34.6%, 35.0%, 39.2%, and 71.0% of the total area in Germany, respectively, while the remaining groups involve more than one hospital, with varying caseload combinations and grouping distances (GD). For groups with near or medium GD—representing 17.7% to 46.8% of all groups—a cooperative policy concept would be highly beneficial. For groups with far GD—accounting for 1.2% to 21.8% of the groups—a special permission with a reduced caseload threshold would help ensure adequate spatial accessibility to the relevant MCR services.

As a supplement to the modelling, the effects of varying the searching distance’s extension step were tested, ranging from 1 min/1 km to 15 min/15 km (Table 5). Using the 1 min/1 km step as the baseline grouping, larger step sizes generally result in more groups being merged. Among all procedures, the modelling for pancreas interventions is most sensitive to the choice of step width. It should be noted that these choices of step size are not a matter of right or wrong; rather, they influence the grouping scale and the proximity differentiation among neighbouring hospitals. This aspect will be further discussed in the Discussion section. The modelling with a 5 min/5 km step, on which the main results are based, is highlighted in bold in Table 5.

**Table 5:** Summary of model sensitivity

	Number of:		Changes to grouping results	affected area size	Group total caseload (GTC) statistics:			
	groups	rounds			Min	Median	Avg.	Max
Oesophagus								
1 min/1 km	57	46	Baseline groups	-	27.9	52.4	78.4	347.6
2 min/2 km	57	24	Identical to baseline groups	0.0%	27.9	52.4	78.4	347.6
3 min/3 km	55	16	4 baseline groups merged into 2; others unchanged	7.2%	27.9	51.4	81.2	347.6
4 min/4 km	55	13	4 baseline groups merged into 2; others unchanged	6.1%	27.9	52.6	81.2	347.6
** 5 min/5 km**	**55**	**10**	**4 baseline groups merged into 2; others unchanged**	**3.0%**	**27.9**	**51.4**	**81.2**	**371.0**
6 min/6 km	52	9	9 baseline groups merged into 4; others unchanged	11.8%	27.9	50.3	85.9	418.0
7 min/7 km	54	8	6 baseline groups merged into 3; others unchanged	12.8%	27.9	51.9	82.7	347.6
8 min/8 km	55	7	4 baseline groups merged into 2; others unchanged	6.1%	27.9	52.6	81.2	347.6
9 min/9 km	51	6	10 baseline groups merged into 4; others unchanged	17.3%	27.9	54.3	87.6	459.2
10 min/10 km	52	6	9 baseline groups merged into 4; others unchanged	12.0%	27.9	52.0	85.9	371.0
11 min/11 km	50	6	13 baseline groups merged into 6; others unchanged	20.9%	27.9	53.4	89.3	371.0
12 min/12 km	48	5	15 baseline groups merged into 6; others unchanged	22.9%	27.9	53.4	93.0	501.6
13 min/13 km	47	5	15 baseline groups merged into 5; others unchanged	22.5%	29.2	51.4	95.0	501.6
14 min/14 km	48	5	13 baseline groups merged into 4; others unchanged	21.8%	27.9	51.9	93.0	501.6
15 min/15 km	47	4	16 baseline groups merged into 6; others unchanged	24.1%	27.9	54.3	95.0	537.6
Pancreas								
1 min/1 km	134	41	Baseline groups	-	20.0	41.6	90.6	879.8
2 min/2 km	131	21	6 baseline groups merged into 3; others unchanged	5.9%	20.0	41.4	92.7	879.8
3 min/3 km	125	15	16 baseline groups merged into 7; others unchanged	18.4%	20.0	40.1	97.1	1095.7
4 min/4 km	126	11	13 baseline groups merged into 5; others unchanged	11.5%	20.0	40.2	96.3	879.8
** 5 min/5 km**	**126**	**9**	**14 baseline groups merged into 6; others unchanged**	**12.4%**	**20.0**	**42.1**	**96.3**	**1413.7**
6 min/6 km	118	8	26 baseline groups merged into 10; others unchanged	25.2%	20.0	40.1	102.9	1165.2
7 min/7 km	115	7	29 baseline groups merged into 10; others unchanged	29.7%	20.0	40.0	105.5	1588.7
8 min/8 km	112	6	32 baseline groups merged into 10; others unchanged	27.8%	20.0	40.1	108.4	1667.4
9 min/9 km	106	6	42 baseline groups merged into 14; others unchanged	37.9%	20.0	39.9	114.5	1707.6
10 min/10 km	112	5	32 baseline groups merged into 10; others unchanged	22.8%	20.0	40.8	108.4	1972.6
11 min/11 km	104	5	44 baseline groups merged into 14; others unchanged	39.9%	20.0	40.8	116.7	1995.3
12 min/12 km	98	5	52 baseline groups merged into 16; others unchanged	45.3%	20.0	41.3	123.9	1486.7
13 min/13 km	94	5	55 baseline groups merged into 15; others unchanged	48.9%	20.0	39.6	129.1	1924.7
14 min/14 km	93	4	55 baseline groups merged into 14; others unchanged	47.2%	20.0	39.9	130.5	1977.6
15 min/15 km	97	4	53 baseline groups merged into 16; others unchanged	48.2%	20.0	40.1	125.1	1907.7
Stem cells								
1 min/1 km	40	33	Baseline groups	-	47.2	182.2	202.6	829.8
2 min/2 km	40	17	Identical to baseline groups	0.0%	47.2	182.2	202.6	829.8
3 min/3 km	40	12	Identical to baseline groups	0.0%	47.2	182.2	202.6	829.8
4 min/4 km	39	9	2 baseline groups merged into 1; others unchanged	5.4%	47.2	192.0	207.8	829.8
** 5 min/5 km**	**39**	**8**	**2 baseline groups merged into 1; others unchanged**	**3.8%**	**47.2**	**192.0**	**207.8**	**829.8**
6 min/6 km	39	7	2 baseline groups merged into 1; others unchanged	3.8%	47.2	192.0	207.8	829.8
7 min/7 km	38	6	4 baseline groups merged into 2; others unchanged	9.3%	47.2	193.8	213.3	829.8
8 min/8 km	39	5	2 baseline groups merged into 1; others unchanged	5.4%	47.2	192.0	207.8	829.8
9 min/9 km	38	5	4 baseline groups merged into 2; others unchanged	9.3%	47.2	193.8	213.3	829.8
10 min/10 km	38	5	4 baseline groups merged into 2; others unchanged	8.4%	47.2	193.8	213.3	829.8
11 min/11 km	38	4	4 baseline groups merged into 2; others unchanged	10.0%	47.2	193.8	213.3	829.8
12 min/12 km	37	4	6 baseline groups merged into 3; others unchanged	13.8%	47.2	195.7	219.0	829.8
13 min/13 km	37	4	6 baseline groups merged into 3; others unchanged	13.3%	47.2	192.0	219.0	829.8
14 min/14 km	37	4	6 baseline groups merged into 3; others unchanged	13.3%	47.2	192.0	219.0	829.8
15 min/15 km	37	4	4 baseline groups merged into 1; others unchanged	5.9%	47.2	172.3	219.0	829.8
Knee								
1 min/1 km	718	42	Baseline groups	-	50.0	124.4	195.4	2355.0
2 min/2 km	711	22	13 baseline groups merged into 6; others unchanged	2.3%	50.0	124.3	197.4	2355.0
3 min/3 km	702	15	28 baseline groups merged into 12; others unchanged	4.3%	50.0	124.2	199.9	2355.0
4 min/4 km	697	12	37 baseline groups merged into 16; others unchanged	7.5%	50.0	125.3	201.3	2355.0
** 5 min/5 km**	**672**	**10**	**72 baseline groups merged into 26; others unchanged**	**13.3%**	**50.0**	**124.4**	**208.8**	**3582.0**
6 min/6 km	681	8	59 baseline groups merged into 22; others unchanged	7.9%	50.0	125.3	206.1	3175.6
7 min/7 km	670	7	75 baseline groups merged into 27; others unchanged	13.3%	50.0	124.9	209.4	3260.0
8 min/8 km	669	7	74 baseline groups merged into 25; others unchanged	14.1%	50.0	125.3	209.8	4610.9
9 min/9 km	665	6	79 baseline groups merged into 26; others unchanged	13.9%	50.0	124.4	211.0	4154.6
10 min/10 km	642	6	105 baseline groups merged into 29; others unchanged	16.9%	50.0	124.4	218.6	4154.6
11 min/11 km	631	5	119 baseline groups merged into 32; others unchanged	19.5%	50.0	124.3	222.4	4309.3
12 min/12 km	614	5	137 baseline groups merged into 33; others unchanged	21.2%	50.0	125.9	228.5	4665.0
13 min/13 km	593	5	158 baseline groups merged into 33; others unchanged	23.2%	50.0	127.4	236.6	4844.8
14 min/14 km	575	4	175 baseline groups merged into 32; others unchanged	23.1%	50.0	127.1	244.1	5055.8
15 min/15 km	566	4	187 baseline groups merged into 35; others unchanged	28.9%	50.0	125.7	247.9	5055.8

The main results of this study are based on models using a 5-min/5-km searching distance extension step, which are highlighted in bold

## Discussion

This study presents a spatial model of hospital cooperation for the joint compliance of MCRs in a multiagent framework. The aim is to apply a cooperation-oriented policy concept to address both MCR caseload compliance and regional accessibility based on the observations of the regional distribution of case numbers related to MCRs. In this model, hospitals are engaged as active stakeholders in the implementation of MCR policy.

### Discussion of results

According to the current distribution of MCR services in Germany, all groups can achieve joint MCR compliance, with a grouping distance of no more than 65 min of driving time. For the four MCR procedures, half or more of the groups consist of only one hospital, which does not require cooperation.

Owing to the significant change in caseload requirements for oesophagus and pancreas procedures, more hospitals in both procedures need special permission with a reduced caseload threshold to ensure spatial accessibility (with long grouping distance, GD) compared with stem cells and knee procedures; this is also true for joint MCR compliance, as the oesophagus and pancreas groups have a greater need for joint compliance (due to medium GD) than do the stem cell and knee procedures. Conversely, the higher proportion of long GD (indicating a recommendation for special permission) and medium GD (indicating a need for joint MCR compliance) in oesophagus and pancreas procedures highlights the need for structural changes to the MCR policy for these procedures. By contrast, stem cell and knee procedures require fewer adjustments.

The modelling results in this study are presented differently from those in previous work [25, 28, 29, 35, 37]. Here, the results are shown as two-dimensional maps and diagrams that incorporate hospital distances (represented by different types of lines) and regional/hospital caseloads (indicated by varying shades of grey) to reflect the MCR-related spatial structure. In contrast, previous studies typically presented these two dimensions – hospital distances and caseloads – independently. Because the main logic in this study is not relocating cases (except in the preparatory round 0), the results are not directly comparable on a one-to-one basis with those of other studies [25, 28, 29, 35, 37]. However, they still align with the general patterns implied by caseload redistribution in earlier work, including the identification of key hospitals within regions that carry most of the cases and those likely to discontinue services and thus require cooperation with nearby hospitals [28, 29]. This similarity is expected, as the resulting caseload distributions are primarily driven by the original spatial distribution of caseloads and the proximity between hospitals, and only to a certain (or lesser) extent by model-specific algorithmic features, such as how regions with low patient density are handled. What distinguishes this study, however, is that it explicitly models hospital participation and presents the policy related parameters in a more visible and discussable form.

### Discussion of methods

Our model introduces a new modelling parameter, ‘group total caseload’, to assess the potential for hospital cooperation, specifically the decision space of hospitals concerning MCR compliance, prior to any policy changes. Unlike other studies that focus on calculating the final distribution of MCR caseloads [25–31], this study places hospitals’ intentions, motivations and decision space at the centre, offering a more nuanced understanding of the potential balance between compliance and service coverage. This approach deliberately avoids directly balancing MCR compliance and accessibility within a centrally governed algorithm. Instead, the balance process is deconstructed into several influencing factors: the regional context, the feasibility of MCR compliance, and the associated decision space of hospitals. Compared with the models [25, 30], which additionally considers maximum travel time and hospital capacity constraints, this study approaches hospital distance and hospital caseload (GTC and GAC) as critical analytical parameters rather than an in-model process parameter. This treatment enables a comprehensive analysis of their function and impact, emphasising how distance and hospital caseload influences hospital cooperation and MCR compliance, including the resulting recommended actions.

According to the model settings, this study defines the thresholds that distinguish cooperating hospitals from noncooperating hospitals at half of the MCR threshold, which can be adjusted. A higher threshold of cooperating hospitals will lead to smoothed zoning edges due to the fewer hospitals as core of Thiessen polygons, but will not affect the general grouping structure and number of groups. The critical proximity boundaries for patient relocation and hospital cooperation, together with the distance threshold for the spatial accessibility of service provision, are key parameters for analysing interrelationships within hospital groups. When policymakers obtain additional medical or social evidence supporting potential improvements, these thresholds can be adjusted to align with specific policy requirements.

The sensitivity analysis of the searching distance extension steps was conducted, and the results are presented in Table 5. Our choice of a 5 min/5 km step is based on the principle that each step should typically aggregate only a small number of hospitals—neither too many at once nor none over extended sequences of steps. Practically, each grouping step represents ‘the next level of proximity’, corresponding to an additional 5 min or 5 km. This policy-oriented modelling approach aims to maintain a spatial scale relevant to cities and city clusters rather than focusing on smaller subdivisions within individual cities.

Two broader considerations should be taken into account when interpreting the modelling approach. First, this study focuses on the broader concept of cooperation and joint compliance with MCRs, rather than on specific forms such as mobile doctors or expertise-sharing. Second, the distances between hospitals are used as the key metric instead of the additional travel distances associated with patient relocation. The former distances provide a rough estimate of the latter.

### Limitations

In this study, federal state boundaries were not considered as an influencing factor or restricting hospital cooperation. In Germany, hospitals are generally not affected by state boundaries, and patients have full freedom to choose hospitals across or within states. Accordingly, state boundaries exert no influence on our model. Furthermore, relationships between hospital sites within the same association were not considered due to the lack of official information on hospital association memberships. Population density and disease prevalence were also not directly addressed, but they were considered indirectly through spatial distribution of hospitals and their caseloads.

## Conclusions

This study employs spatial modelling to examine MCR compliance at the regional level, exploring the novel concept of joint MCR compliance and potential hospital cooperation, including the formation, categorisation, and characteristics of hospital groups. The construction of this model enables the testing and analysis of varying policy agreements by defining parameter thresholds. This approach provides operational flexibility for hospitals. It also serves as a theoretical basis for granting exceptional MCR permissions to achieve spatial accessibility of service provision.

## Supplementary Information

Supplementary Material 1

## Data Availability

The GHQR data for this study are available from the Federal Joint Committee upon reasonable request. The processed data will be shared upon request with the corresponding author with permission from the Federal Joint Committee.

## Abbreviations

GAC: The group average caseload per cooperating hospital
GD: Grouping distance
GHQR: German hospital quality reports
GTC: Group total caseload
MCR: Minimum caseload requirements
SLD: Straight-line distance

## References

[1] Luft HS, Bunker JP, Enthoven AC. Should operations be regionalized? The empirical relation between surgical volume and mortality. N Engl J Med. 1979;301:1364–9. 10.1056/NEJM197912203012503. 503167 10.1056/NEJM197912203012503

[2] Luft HS. The relation between surgical volume and mortality: an exploration of causal factors and alternative models. Med Care. 1980;18:940–59. 10.1097/00005650-198009000-00006. 7432019 10.1097/00005650-198009000-00006

[3] Birkmeyer JD, Siewers AE, Finlayson EVA, Stukel TA, Lucas FL, Batista I, et al. Hospital volume and surgical mortality in the united States. N Engl J Med. 2002;346:1128–37. 10.1056/NEJMsa012337. 11948273 10.1056/NEJMsa012337

[4] Birkmeyer JD. Strategies for improving surgical quality–checklists and beyond. N Engl J Med. 2010;363:1963–5. 10.1056/NEJMe1009542. 21067390 10.1056/NEJMe1009542

[5] Pieper D, Mathes T, Neugebauer E, Eikermann M. State of evidence on the relationship between high-volume hospitals and outcomes in surgery: a systematic review of systematic reviews. J Am Coll Surg. 2013;216:1015-1025.e18. 10.1016/j.jamcollsurg.2012.12.049. 23528183 10.1016/j.jamcollsurg.2012.12.049

[6] Levaillant M, Marcilly R, Levaillant L, Michel P, Hamel-Broza J-F, Vallet B, Lamer A. Assessing the hospital volume-outcome relationship in surgery: a scoping review. BMC Med Res Methodol. 2021;21:204. 10.1186/s12874-021-01396-6. 34627143 10.1186/s12874-021-01396-6 PMC8502281

[7] de Cruppé W, Malik M, Geraedts M. Achieving minimum caseload requirements: an analysis of hospital quality control reports from 2004–2010. Dtsch Arztebl Int. 2014;111:549–55. 10.3238/arztebl.2014.0549. 25220064 10.3238/arztebl.2014.0549 PMC4165182

[8] Morche J, Mathes T, Pieper D. Relationship between surgeon volume and outcomes: a systematic review of systematic reviews. Syst Rev. 2016;5:204. 10.1186/s13643-016-0376-4. 27899141 10.1186/s13643-016-0376-4 PMC5129247

[9] Morche J, Renner D, Pietsch B, Kaiser L, Brönneke J, Gruber S, Matthias K. International comparison of minimum volume standards for hospitals. Health Policy. 2018;122:1165–76. 10.1016/j.healthpol.2018.08.016. 30193981 10.1016/j.healthpol.2018.08.016

[10] Vonlanthen R, Lodge P, Barkun JS, Farges O, Rogiers X, Soreide K, et al. Toward a consensus on centralization in surgery. Ann Surg. 2018;268:712–24. 10.1097/SLA.0000000000002965. 30169394 10.1097/SLA.0000000000002965

[11] Polonski A, Izbicki JR, Uzunoglu FG. Centralization of pancreatic surgery in Europe. J Gastrointest Surg. 2019;23:2081–92. 10.1007/s11605-019-04215-y. 31037503 10.1007/s11605-019-04215-y

[12] Fong ZV, Loehrer AP, Fernández-Del Castillo C, Bababekov YJ, Jin G, Ferrone CR, et al. Potential impact of a volume pledge on spatial access: a population-level analysis of patients undergoing pancreatectomy. Surgery. 2017;162:203–10. 10.1016/j.surg.2017.03.010. 28504112 10.1016/j.surg.2017.03.010 PMC5889083

[13] Resio BJ, Chiu AS, Hoag JR, Brown LB, White M, Omar A, et al. Motivators, barriers, and facilitators to traveling to the safest hospitals in the United States for complex cancer surgery. JAMA Netw Open. 2018;1:e184595. 10.1001/jamanetworkopen.2018.4595. 30646367 10.1001/jamanetworkopen.2018.4595 PMC6324377

[14] Sheetz KH, Chhabra KR, Smith ME, Dimick JB, Nathan H. Association of discretionary hospital volume standards for high-risk cancer surgery with patient outcomes and access, 2005–2016. JAMA Surg. 2019;154:1005–12. 10.1001/jamasurg.2019.3017. 31411663 10.1001/jamasurg.2019.3017 PMC6694402

[15] Burkamp J, Bühn S, Pieper D. Patientenpräferenz Im Spannungsfeld Zwischen mindestmengen und flächendeckender versorgung am beispiel der Knietotalendoprothese. [Patient preferences between minimum volume thresholds and nationwide healthcare provision: the example of total knee Arthroplasty]. Z Orthop Unfall. 2020;158:390–6. 10.1055/a-0965-7720. 31525791 10.1055/a-0965-7720

[16] Daskin MS, Dean LK. Location of health care facilities. In: Operations research and health care: A handbook of methods and applications. 2004. p. 43–76.

[17] Hamid A, Peng Q. Challenges and solutions for location of healthcare facilities. Ind Eng Manage. 2014. 10.4172/2169-0316.1000127.

[18] Ahmadi-Javid A, Seyedi P, Syam SS. A survey of healthcare facility location. Comput Oper Res. 2017;79:223–63. 10.1016/j.cor.2016.05.018.

[19] Laporte G, Nickel S, Da Saldanha Gama F. Location science. Cham: Springer International Publishing; 2019.

[20] Listorti E, Alfieri A, Pastore E. Hospital volume allocation: integrating decision maker and patient perspectives. Health Care Manag Sci. 2022;25:237–52. 10.1007/s10729-021-09586-w. 34709503 10.1007/s10729-021-09586-w PMC9165272

[21] Burdett RL, Corry P, Yarlagadda P, Cook D, Birgan S, McPhail SM. A mathematical framework for regional hospital case mix planning and capacity appraisal. Oper Res Perspect. 2023;10:100261. 10.1016/j.orp.2022.100261.

[22] Bruno G, Cavola M, Diglio A, Piccolo C. Improving spatial accessibility to regional health systems through facility capacity management. Socio-Econ Plann Sci. 2020;71:100881. 10.1016/j.seps.2020.100881.

[23] Fränti P, Mariescu-Istodor R, Akram A, Satokangas M, Reissell E. Can we optimize locations of hospitals by minimizing the number of patients at risk? BMC Health Serv Res. 2023;23:415. 10.1186/s12913-023-09375-x. 37120539 10.1186/s12913-023-09375-x PMC10148542

[24] Oliveira MD, Bevan G. Modelling the redistribution of hospital supply to achieve equity taking account of patient’s behaviour. Health Care Manag Sci. 2006;9:19–30. 10.1007/s10729-006-6277-7. 16613014 10.1007/s10729-006-6277-7

[25] Mennicken R, Kolodziej IWK, Augurzky B, Kreienberg R. Concentration of gynaecology and obstetrics in Germany: is comprehensive access at stake? Health Policy. 2014;118:396–406. 10.1016/j.healthpol.2014.07.017. 25201487 10.1016/j.healthpol.2014.07.017

[26] Hentschker C, Mennicken R. The volume-outcome relationship and minimum volume standards–empirical evidence for Germany. Health Econ. 2015;24:644–58. 10.1002/hec.3051. 24700615 10.1002/hec.3051

[27] Leber W-D, Scheller-Kreinsen D. Marktaustritte sicherstellen-Zur Rolle rekursiver Simulationen Bei der Strukturbereinigung Im Krankenhaussektor [Ensuring market exits: the role of recursive simulations in structural adjustments in the hospital sector]. In: Klauber J, Geraedts M, Friedrich J, Wasem J, editors. Krankenhaus-Report 2015 [hospital report 2015]. Stuttgart: Schattauer; 2015. p. 187–210.

[28] Hentschker C, Mennicken R, Reifferscheid A, Wasem J, Wübker A. Volume-outcome relationship and minimum volume regulations in the German hospital sector - evidence from nationwide administrative hospital data for the years 2005–2007. Health Econ Rev. 2018;8:25. 10.1186/s13561-018-0204-8. 30259207 10.1186/s13561-018-0204-8 PMC6755587

[29] IQTIG. Folgenabschätzungen zu Mindestmengen. [Impact assessments of minimum volume standards]. 2021. https://iqtig.org/veroeffentlichungen/folgenabschaetzungen-mm/

[30] Vogel JFA, Barkhausen M, Pross CM, Geissler A. Defining minimum volume thresholds to increase quality of care: a new patient-oriented approach using mixed integer programming. Eur J Health Econ. 2022. 10.1007/s10198-021-01406-w. 35089456 10.1007/s10198-021-01406-w PMC9395474

[31] Hansis D, Dahnke H. Wie mindestmengen die patientenwanderung beeinflussen. [The influence of minimum volume requirements on patient migration]. MVF. 2024;17:51–4. 10.24945/MVF.01.24.1866-0533.2579.

[32] Knappich C, Tsantilas P, Salvermoser M, Schmid S, Kallmayer M, Trenner M, et al. Editor’s choice - distribution of care and hospital incidence of carotid endarterectomy and carotid artery stenting: a secondary analysis of German hospital episode data. Eur J Vasc Endovasc Surg. 2021;62:167–76. 10.1016/j.ejvs.2021.03.021. 33966984 10.1016/j.ejvs.2021.03.021

[33] Spangenberg M. Erreichbarkeit von Krankenhäusern [Accessibility of Hospitals]. In: Klauber J, Geraedts M, Friedrich J, Wasem J, editors. Krankenhaus-Report 2012 [hospital report 2012]. Stuttgart: Schattauer; 2012. p. 97–109.

[34] Versteeg SE, Ho VKY, Siesling S, Varkevisser M. Centralisation of cancer surgery and the impact on patients’ travel burden. Health Policy. 2018;122:1028–34. 10.1016/j.healthpol.2018.07.002. 30060899 10.1016/j.healthpol.2018.07.002

[35] Deutschland Bundesministerium des Innern, für Bau und Heimat. Deutschlandatlas: Karten zu gleichwertigen Lebensverhältnissen [Germany Atlas: Maps on equal living conditions]. 2019. https://www.bmleh.de/SharedDocs/Downloads/DE/_laendliche-Regionen/deutschlandatlas2019.html

[36] Mediqon M. Wie beeinflussen Mindestmengenvorgaben die Erreichbarkeit in der stationären Versorgung? [Minimum quantities: How do minimum volume requirements affect accessibility in inpatient care?]. 2024. https://mediqon.de/mindestmengen/

[37] Bauer J, Klingelhöfer D, Maier W, Schwettmann L, Groneberg DA. Spatial accessibility of general inpatient care in germany: an analysis of surgery, internal medicine and neurology. Sci Rep. 2020;10:19157. 10.1038/s41598-020-76212-0. 33154470 10.1038/s41598-020-76212-0 PMC7645718

[38] World Health Organization. Strategizing National health in the 21st century: a handbook. World Health Organization; 2016.

[39] Cliff AD, Ord JK. Model building and the analysis of spatial pattern in human geography. J Roy Stat Soc: Ser B (Methodol). 1975;37:297–328. 10.1111/j.2517-6161.1975.tb01548.x.

[40] Griffith DA, Jones KG. Explorations into the relationship between spatial structure and spatial interaction. Environ Plan A. 1980;12:187–201. 10.1068/a120187.

[41] Tong D, Murray AT. Spatial optimization in geography. Ann Assoc Am Geogr. 2012;102:1290–309. 10.1080/00045608.2012.685044.

[42] Federal Joint Committee (Gemeinsamer Bundesausschuss, G-BA). German Hospital Quality Report 2016, 2017, 2018, 2019, 2020, 2021. https://www.g-ba.de/themen/qualitaetssicherung/datenerhebung-zur-qualitaetssicherung/datenerhebung-qualitaetsbericht/

[43] Ji L, Geraedts M, de Cruppé W. Internal validation of self-reported case numbers in hospital quality reports: preparing secondary data for health services research. BMC Med Res Methodol. 2024;24:325. 10.1186/s12874-024-02429-6. 39736545 10.1186/s12874-024-02429-6 PMC11686984

[44] Ji L, Geraedts M, de Cruppé W. A theoretical framework for linking hospitals longitudinally: demonstrated using German hospital quality reports 2016–2020. BMC Med Res Methodol. 2024;24:212. 10.1186/s12874-024-02317-z. 39300394 10.1186/s12874-024-02317-z PMC11411731

[45] Google GM, Geocoding API. 2023. https://developers.google.com/maps/documentation/geocoding

[46] Battiston C. GeoPy (Version 2.3.0) [Python Library]. 2023. https://geopy.readthedocs.io/

[47] Heidelberg Institute for Geoinformation Technology. Openrouteservice, 2024. https://openrouteservice.org/

[48] Esri. ArcGIS Pro (Version 2024) [Software]. Environmental Systems Research Institute. 2024. https://www.esri.com

[49] Federal Joint Committee (Gemeinsamer Bundesausschuss, G-BA). Regelungen des Gemeinsamen Bundesausschusses gemäß § 136b Absatz 1 Satz 1 Nummer 2 SGB V für nach § 108 SGB V zugelassene Krankenhäuser. (Mindestmengenregelungen, Mm-R). [Minimum Volume Regulations]. 2024. https://www.g-ba.de/richtlinien/5/historie/

[50] GADM. GADM database (Version 2.5) [Global Administrative Areas Database]. 2024. https://www.gadm.org

