# Supplementary material for: Cooperative spatial modelling of hospital compliance with minimum caseload requirements
Source: Int J Health Geogr. 2026 Jan 27;25:9. doi: 10.1186/s12942-025-00442-6 (PMC12866498; doi:10.1186/s12942-025-00442-6)
Supplement: Supplementary file 1 — Supplementary Material 1 [file 12942_2025_442_MOESM1_ESM.docx]

**Supplementary material**


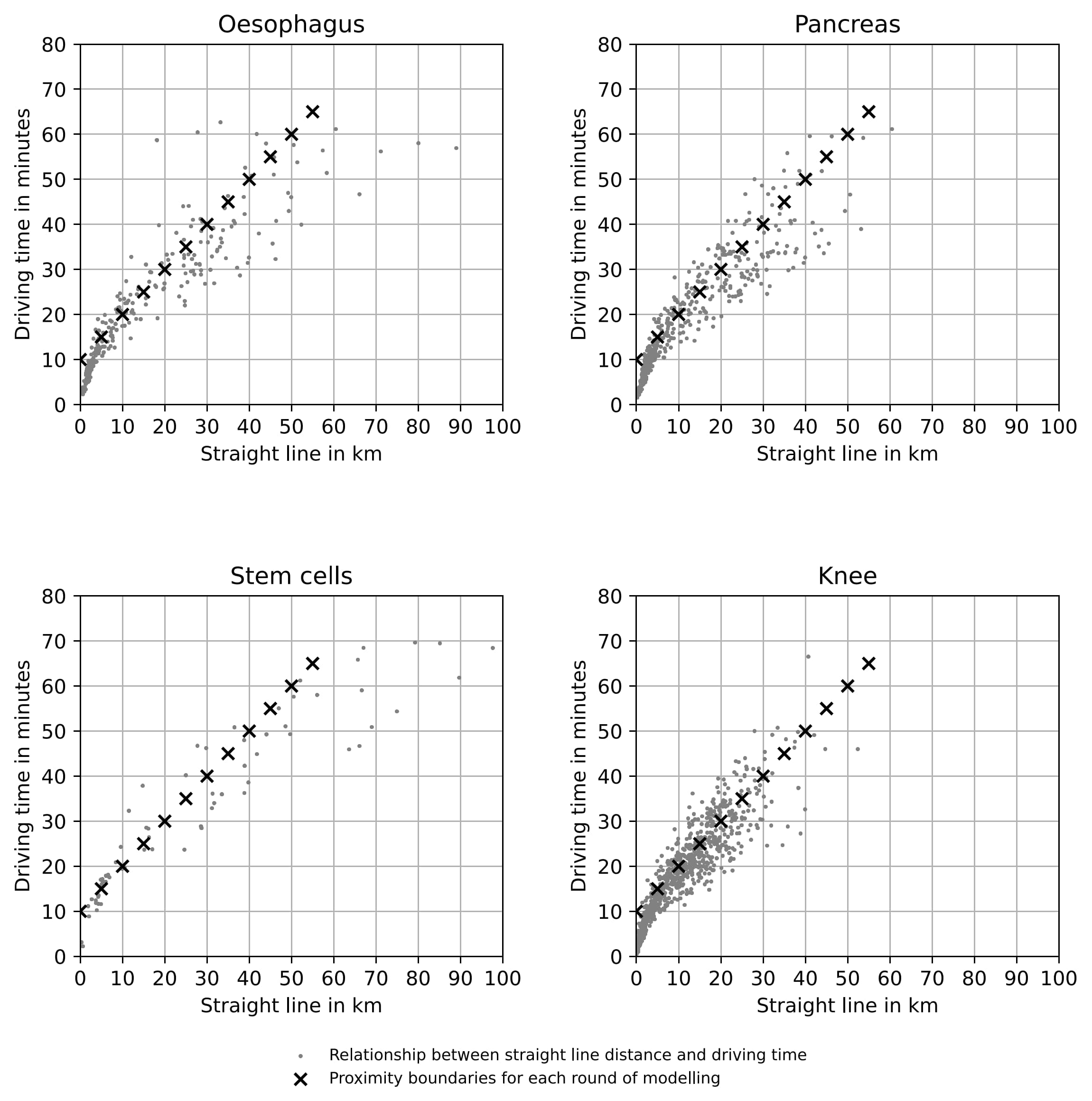


**Figure S1**: Relationships between two types of distance: straight line distance (x-axis) and driving time (y-axis): the distance from the cooperating hospital to the nearest cooperating hospital.
